# Hospital Festivals and Meetings

**Published:** 1894-06-23

**Authors:** 


### HOSPITAL FESTIVALS AND MEETINGS.

### BELGRAVE HOSPITAL FOR CHILDREN.

A very well-attended meeting was held on June 13th at this hospital, the Duke of Westminster presiding. Unfortunately the institution is considerably in debt owing in great measure to the necessity of remodelling the drainage system last year; and the committee have under their consideration the desirability of closing one of the wards, a step which they hope, however, to be enabled to avoid taking. Mr. C. T. Dent, in moving the appointment of a Ladies' Committee to aid in the work of the hospital, said he thought that their institution suffered from excessive modesty, and did not make itself well enough known. It was, however, widely known amongst the poor, and patients came to it from such distant parts as Battersea. Other speakers were Sir John Tilley, Mr. Thomas, Dr. Ewart, and Colonel Maxwell, who moved a vote of thanks to Miss Palmes, the lady superintendent. The business of the meeting being over, the Duke and Duchess of Westminster went to inspect the wards and to declare one of them—the Princess Beatrice Ward—formally opened. The number of in-patients last year was 225, an increase of 29 over 1892; and of out-patients 4,576, also a large increase over the numbers of the year before.

